# Pooling data on different probiotics is not appropriate to assess the efficacy of probiotics

**DOI:** 10.1007/s00431-014-2340-4

**Published:** 2014-05-22

**Authors:** Hania Szajewska

**Affiliations:** Department of Paediatrics, The Medical University of Warsaw, Dzialdowska 1, 01-184 Warsaw, Poland

Dear Editor,

Li et al. [2] presented the pooled results of randomized controlled trials and concluded that probiotic administration increases the *H pylori* eradication rate and reduces the risk of therapy-related side effects.

It is tempting to produce a single estimate of the treatment effect of probiotics. However, pooling data from different strains and doses of probiotics obtained in different settings and/or populations may result in misleading conclusions. The risk is that the results could be erroneously extrapolated to other probiotics. This concern is shared by many experts in the field of probiotics [1, 3, 4].

In settings where there are tens of different probiotic products available, one wants to know the efficacy of one specific probiotic, not of probiotics in general. The meta-analysis by Li et al. does not help to resolve such uncertainty.

What could be the solution? One approach could be to perform a meta-analysis that evaluates the effect of administering a clearly defined, probiotic(s). In children, for there is no single probiotic that has been studied in more than one RCT. With a lack of repeat studies, can we be sure that all or any probiotic(s) studied are really effective?

In my opinion, those of us who are working in the probiotic field should avoid pooling data on different probiotics.
